# Supplementary material for: Learning deep representations of enzyme thermal adaptation
Source: Protein Sci. 2022 Dec;31(12):e4480. doi: 10.1002/pro.4480 (PMC9679980; doi:10.1002/pro.4480)
Supplement: Supplementary file 1 — APPENDIX S1 Supporting Information [file PRO-31-e4480-s001.pdf]

Supplementary information for

Learning deep representations of enzyme thermal adaptation

Gang Li[1#], Filip Buric[1#], Jan Zrimec[1,2], Sandra Viknander[1], Jens Nielsen[1,3], Aleksej Zelezniak[1,4] and Martin KM Engqvist[1,*]

[1] Department of Biology and Biological Engineering, Chalmers University of Technology, Kemivägen 10, SE-412 96 Gothenburg, Sweden

[2] Department of Biotechnology and Systems Biology, National Institute of Biology, Večna pot 111, SI-1000 Ljubljana, Slovenia

[3] BioInnovation Institute, Ole Måløes Vej 3, DK-2200 Copenhagen N, Denmark

[4] Institute of Biotechnology, Life Sciences Centre, Vilnius University, Sauletekio al. 7, LT-10257 Vilnius, Lithuania

# Equal contribution

* Corresponding author

E-mail: martin.engqvist@chalmers.se

Table of contents

Supplementary figures … p.2 - p.10

Supplementary tables … p.10 - p.14

Supplementary references … p.15

Supplementary Figures

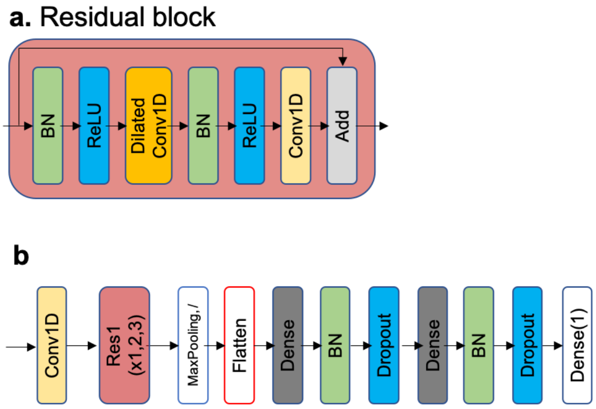

Figure S1. The ResNet based model architecture used in this study. (a) the residual block, taken from (*1*) (b) the model architecture. It takes one-hot encoded protein sequences as input. The number of residual blocks (Res1) was treated as a hyperparameter varying from 1-3.

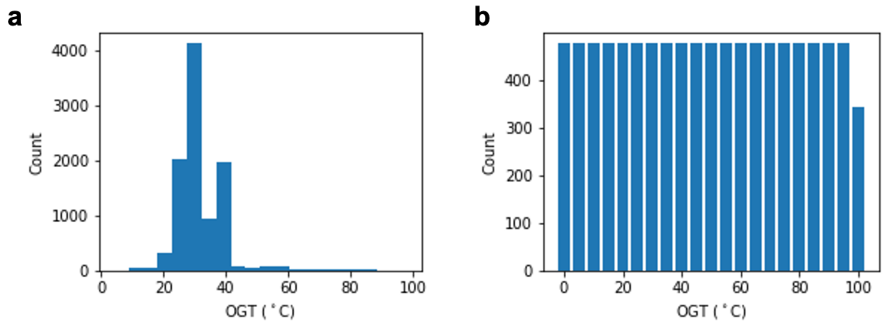

Figure S2. OGT datasets for hyper-parameter optimization. (a) the distribution of OGT values of enzymes randomly sampled from the original training dataset. (b) a uniformly distributed dataset was sampled from the original training dataset. There are 10,000 enzymes in each dataset.

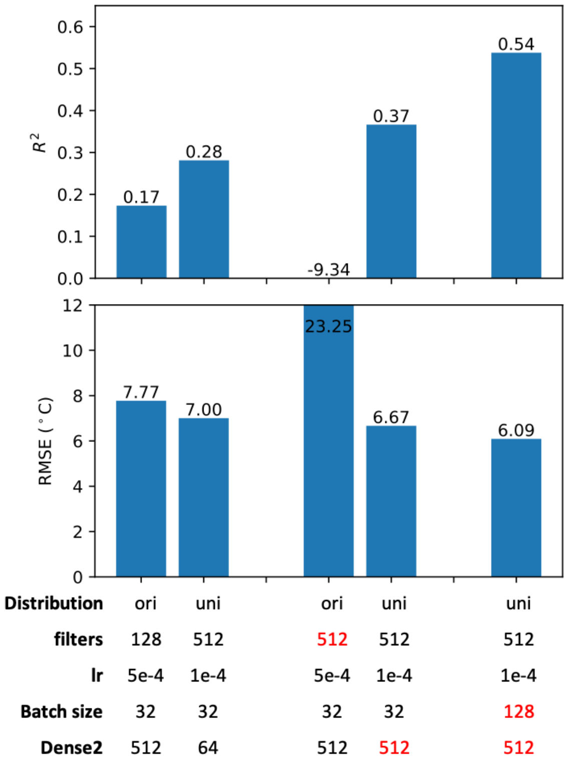

Figure S3. Validation metrics from hyper-parameter tuning results. The upper and lower panel are the R2 score and root mean squared error on the validation dataset. The description under the figure is variables tuned. **Distribution**, if the hyper-parameter set is achieved on a dataset with original distribution (ori) or uniform distribution as shown in Figure S2; **filters**, the number of filters used in all convolution layers; **lr**, learning rate; **Dense2**, the number of nodes in the second fully-connected layer (Figure S1). The hyper-parameter sets of the first two bars were obtained by a random search approach on two datasets as shown in Figure S2. Then the number of filters and the size of the Dense2 were increased to 512, respectively (3rd and 4th bar). The bad scores shown in the 3rd bar were due to the training not being finished in 7-days. Since the model architecture optimised on the uniformly distributed dataset shows better performance on big OGT validation dataset, it was further tuned by increasing the **batch size** from 32 to 128. In the end, the hyper-parameters used for the last bar were considered as the final hyper-parameters (Detailed list is in Table S2).

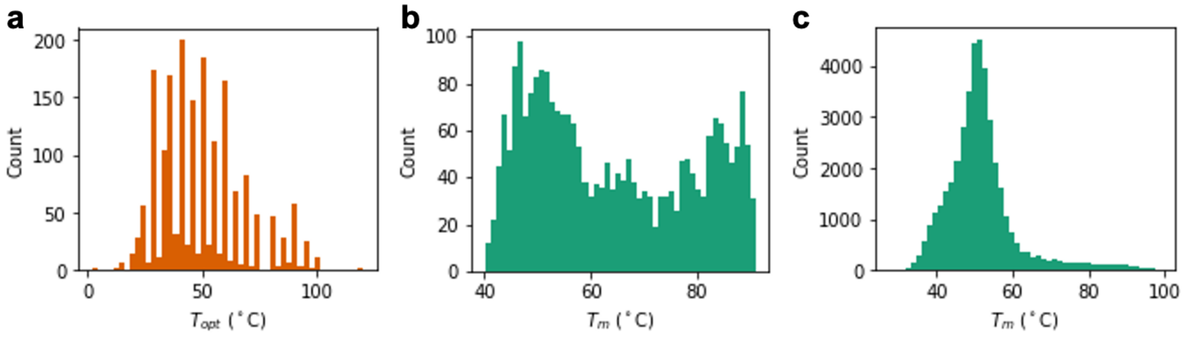

Figure S4. Distributions of (a) enzyme T_{opt} from (*2*), protein melting temperatures from Leuenberger P et al (*3*) and Jarzab A et al (*4*). See details in the Methods section.

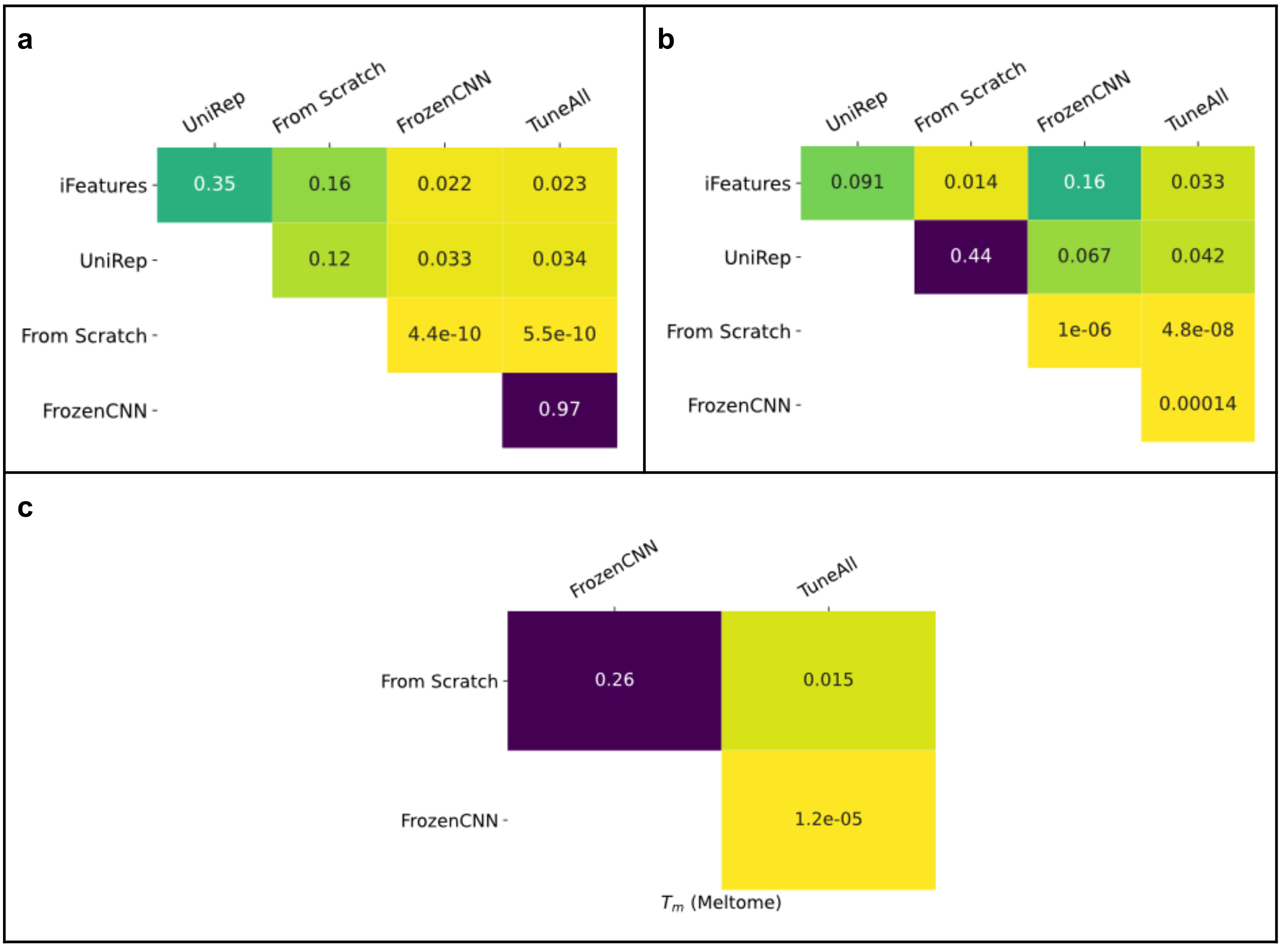

Figure S5. Significance of model performance differences. Welch's t-test was performed on R^2 measures of the different respective model training iterations. The matrices show p-values for the (a) TOPT test dataset (b) TM test dataset (c) MELT test dataset, matching Figure 2a-c.

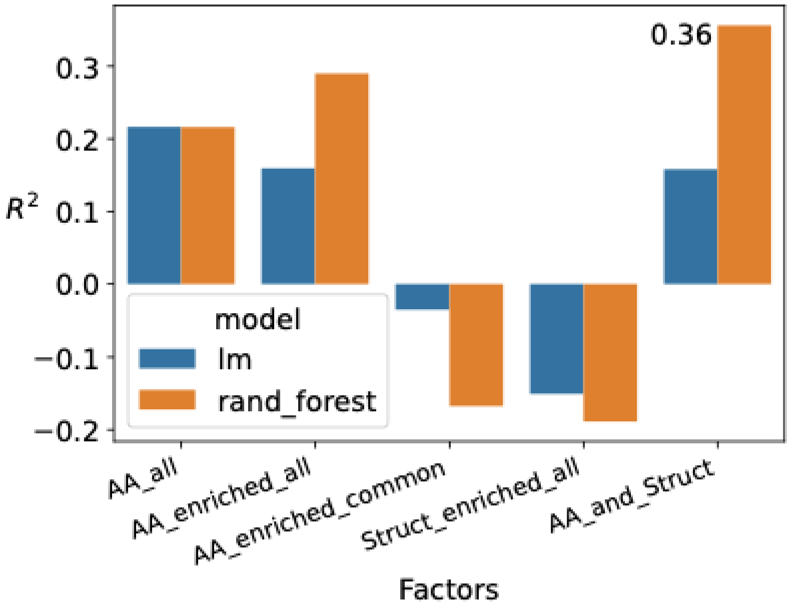

Figure S6. Predictivity of the determinative sequence features identified through perturbation of DeepET T_{opt} predictions. The linear (*lm*) and random forest (*rand_forest*) models were trained and evaluated on the same train-test split of the TM dataset as for the deep models (Fig. 2). The combinations of model factors are: *AA_all* = the composition of all amino acids, *AA_enriched_all* = the composition of all enriched amino acids in the sequence relevance profiles, *AA_enriched_common* = the composition of the common enriched amino acids in the sequence relevance profiles of mesophiles and thermophiles, *Struct_enriched_all* = the composition of all enriched secondary structures in the sequence relevance profiles, *AA_and_Struct* = combination of *AA_enriched_all* and *Struct_enriched_all*. The highest performance (R^2 = 0.36, RMSE = 16) was obtained for a random forest model trained on all enriched factors (*AA_and_Struct*).

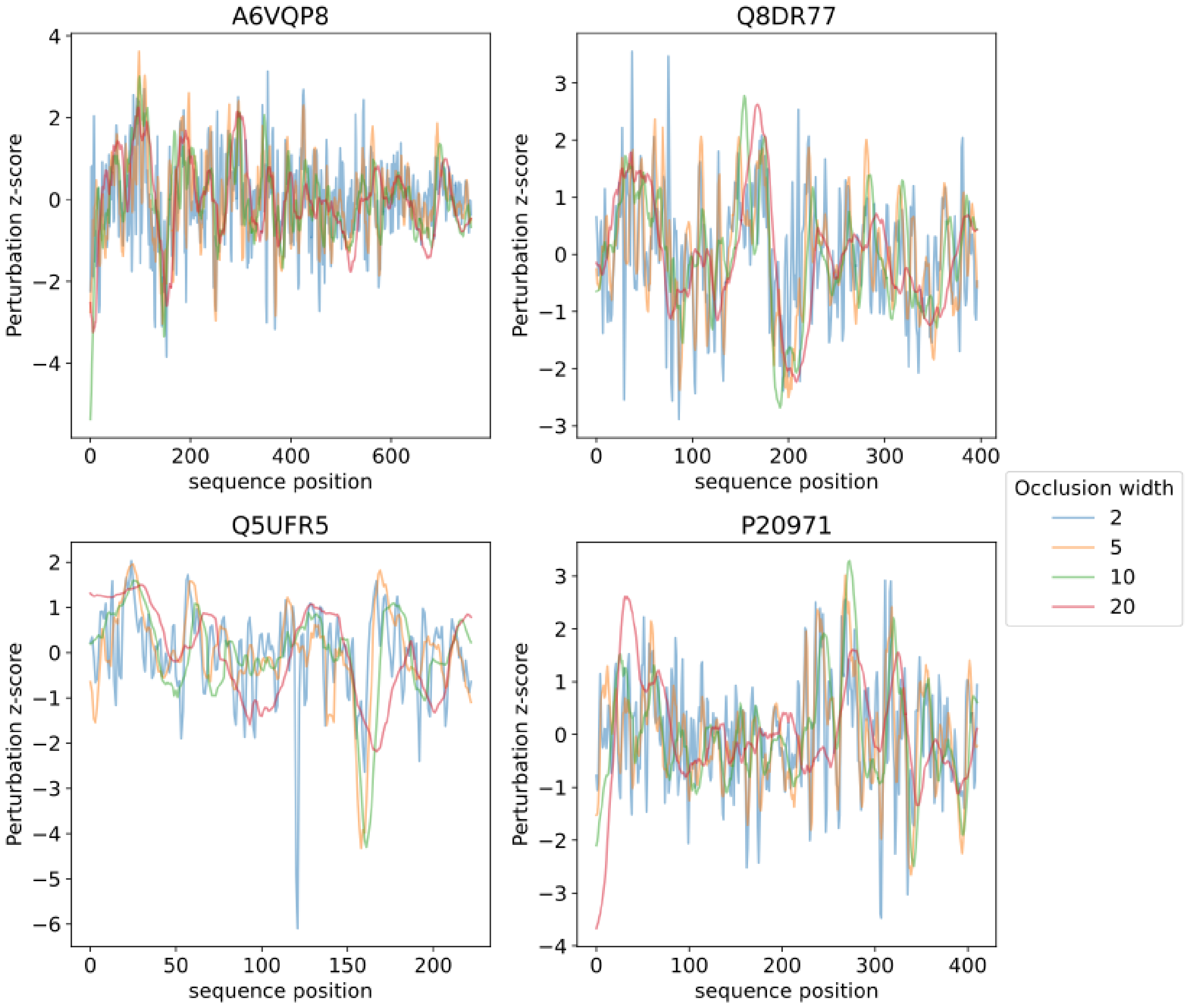

Figure S7. Comparison of perturbation profiles using different occlusion widths, for 4 randomly selected sequences (UniProt IDs given as subfigure titles), showing the overall large similarity when using different widths.

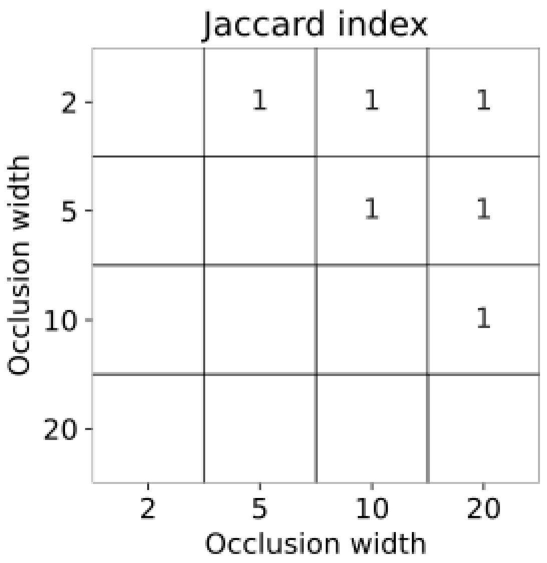

Figure S8. The choice of occlusion window width has no impact on the set of protein domains covered by significant perturbation profile positions. Indeed, the sets resulting from different occlusion window widths overlap perfectly, as shown here by the Jaccard index of 1 between these (showing only the upper triangle, as this measure is symmetric).

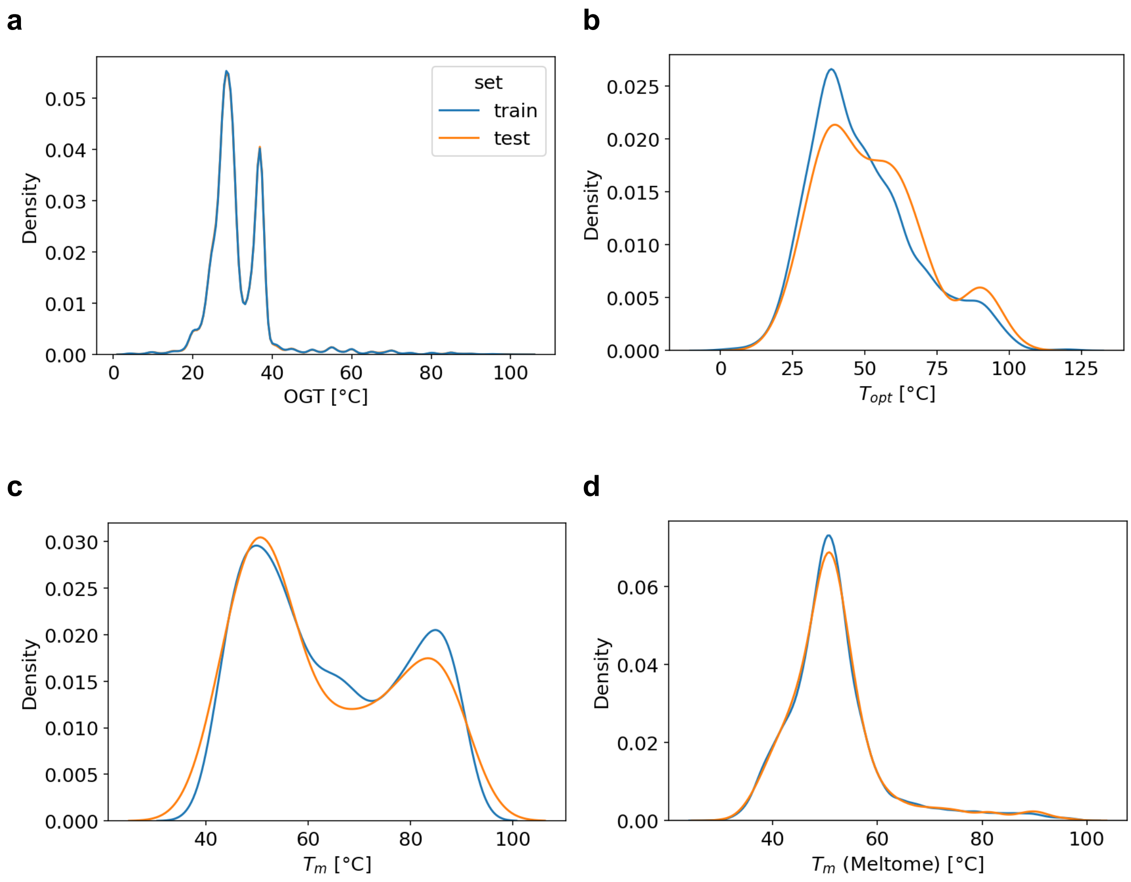

Figure S9. Temperature distribution comparison between training and test subsets of the (a) OGT, (b) TOPT, (c) TM, and (d) MELT datasets. The random split of training and test sets preserved the overall distribution between these two subsets. OGT subsets were subsampled (uniformly) to 5e5 values each, to avoid numerical issues in the kernel density estimate calculation.

Supplementary Tables

Table S1. Hyper parameter space used for random search search

**Name**	**Space**	**Applied to layers**
filters	[32, 64, 128, 256, 512]	All convolution layers have the same number of filters
kernel size	[3, 7, 9, 11, 21, 31]	All convolution layers, they have different kernel sizes
dilation (ResBlock)	[1, 2, 3, 5]	The first conv1d layer in ResBlock
pool size	[2, 4, 8, 20, 30, 40]	The max pooling layer, pool size is equal to strides
Dense 1	[256, 512, 1024]	First dense layer
Dense 2	[128, 256, 512]	Second dense layer
Dropout	(0,0.5)	Two dense layers, they have different dropout values
lr	[1e-4, 5e-4, 1e-3]	
mbatch	[32, 64, 128, 256]	
Number of Residual blocks	[1, 2, 3]	

Table S2. Optimised hyper-parameters

		OriDist (Figure S2a)	UniDist(Figure S2b)
	filters	128	512
	Kernel size 1	7	9
Residual Block 1	Kernel size 21	7	21
	Kernel size 22	7	11
	dilation2	1	1
Residual Block 2	Kernel size 31	31	NA
	Kernel size 32	21	NA
	dilation3	3	NA
	Pool size (=strides)	30	50
	dense1	512	512
	Drop out 1	0.35	0.17
	dense2	512	64 → **512**
	Drop out 2	0.37	0.15
	lr	5e-4	1e-4
	mbatch	32	32 → **128**
	Total weights	5.8M	19.0M → 19.2M

Table S3. Biological process GO slims for the most relevant domains for T_{opt} prediction of mesophilic and thermophilic enzymes.

GO ID	Mesophiles	Thermophiles
GO:0009058	biosynthetic process	
GO:0044281	small molecule metabolic process	small molecule metabolic process
GO:0006259	DNA metabolic process	DNA metabolic process
GO:0002376	immune system process	
GO:0007155	cell adhesion	
GO:0034641	cellular nitrogen compound metabolic process	
GO:0006605	protein targeting	
GO:0009056	catabolic process	catabolic process
GO:0015031	protein transport	
GO:0005975	carbohydrate metabolic process	carbohydrate metabolic process
GO:0006091	generation of precursor metabolites and energy	
GO:0006950	response to stress	response to stress
GO:0006629	lipid metabolic process	
GO:0006520	cellular amino acid metabolic process	cellular amino acid metabolic process
GO:0006399		tRNA metabolic process

Table S4. Molecular function GO slims for the most relevant domains for T_{opt} prediction of mesophilic and thermophilic enzymes.

GO ID	Mesophiles	Thermophiles
GO:0016491	oxidoreductase activity	oxidoreductase activity
GO:0016779	nucleotidyltransferase activity	
GO:0016791	phosphatase activity	phosphatase activity
GO:0016301	kinase activity	kinase activity
GO:0008233	peptidase activity	peptidase activity
GO:0016829	lyase activity	
GO:0016765	transferase activity, transferring alkyl or aryl (other than methyl) groups	
GO:0043167	ion binding	ion binding
GO:0003677	DNA binding	
GO:0016798	hydrolase activity, acting on glycosyl bonds	hydrolase activity, acting on glycosyl bonds
GO:0016810	hydrolase activity, acting on carbon-nitrogen (but not peptide) bonds	
GO:0008168		methyltransferase activity
GO:0016874		ligase activity
GO:0016853		isomerase activity

Supplementary References

1. M. L. Bileschi, D. Belanger, D. Bryant, T. Sanderson, B. Carter, D. Sculley, M. A. DePristo, L. J. Colwell, Using Deep Learning to Annotate the Protein Universe. *bioRxiv* (2019), p. 626507.

2. G. Li, J. Zrimec, B. Ji, J. Geng, J. Larsbrink, A. Zelezniak, J. Nielsen, M. K. M. Engqvist, Performance of regression models as a function of experiment noise. *arXiv [q-bio.BM]* (2019), (available at http://arxiv.org/abs/1912.08141).

3. P. Leuenberger, S. Ganscha, A. Kahraman, V. Cappelletti, P. J. Boersema, C. von Mering, M. Claassen, P. Picotti, Cell-wide analysis of protein thermal unfolding reveals determinants of thermostability. *Science*. **355** (2017), doi:10.1126/science.aai7825.

4. A. Jarzab, N. Kurzawa, T. Hopf, M. Moerch, J. Zecha, N. Leijten, Y. Bian, E. Musiol, M. Maschberger, G. Stoehr, I. Becher, C. Daly, P. Samaras, J. Mergner, B. Spanier, A. Angelov, T. Werner, M. Bantscheff, M. Wilhelm, M. Klingenspor, S. Lemeer, W. Liebl, H. Hahne, M. M. Savitski, B. Kuster, Meltome atlas-thermal proteome stability across the tree of life. *Nat. Methods* (2020), doi:10.1038/s41592-020-0801-4.
